# Automated Internal Classification of Beadless Chinese ZhuJi Fleshwater Pearls based on Optical Coherence Tomography Images

**DOI:** 10.1038/srep33819

**Published:** 2016-09-26

**Authors:** Yang Zhou, Tiebing Liu, Yang Shi, Zhengwei Chen, Jianwei Mao, Wujie Zhou

**Affiliations:** 1 School of Information and Electronic Engineering, Zhejiang University of Science and Technology, Hangzhou, 310023, China; 2 Institute Zhejiang Provincial key Lab for Chem&Bio Processing Technology of Farm Product, Hangzhou, 310023, China; 3 Center of Engineering Training, Zhejiang University of Science and Technology, Hangzhou, 310023, China

## Abstract

Optical coherence tomography (OCT) has been applied to inspect the internal defect of beadless Chinese ZhuJi fleshwater pearls. A novel fully automated algorithm is proposed to classify between normal and defective sub-layer in nacre layer. Our algorithm utilizes the graph segmentation approach to estimate the up and down boundaries of defect sub-layers from flattened and cropped image, and also proposes the strategy for edge and weight construction in segmentation process. The vertical gradients of boundary pixels are used to make grading decision. The algorithm is tested by typical pearl samples, and achieves 100% classification accuracy. The experiment result shows the feasibility and adaptability of the proposed approach, and proves that the OCT technique combined with proposed algorithm is a potential tool for fast and non-destructive diagnosis of internal structure of beadless pearl.

Pearl is one of the beautiful gemstones and gains recognition by worldwide market and customers^1^. China ZhuJi is a main producing area of fleshwater pearl, and it constitutes about 90% of pearl production in China^2^. Because of huge yields of fleshwater pearl, the pearl quality inspection has been paid more attention. The internal inspection includes shape, luster and surface defect which can be evaluated by manual and automated testing^3^,^4^, however, the internal quality inspection is still a difficult point, especially for non-destructive evaluation.

As a non-destructive internal imaging method, optical coherence tomography (OCT) has been tried to compete the task. OCT technique is an effective noninvasive technique for acquiring the microstructure information at the resolution of 10 micrometer^5^,^6^. It can be easily used for classing between the beaded and non-beaded pearls, detecting the internal defect of pearls, identifying the pearls’ nuclei, measuring the nacre thickness of beaded pearls, and so on ref. 7, 8, 9. Combing with fluorescence spectroscopy, it is possible to use the OCT system for pretreatment or treatment identification of commercial pearls^10^. In addition to nondestructive, OCT technique for pearls has many other advantages, including high scan speed, easy operation and strong adaptability of almost all kinds of pearls.

Most of pearls cultured inland region of ZhuJi are beadless fleshwater pearls. The nacreous layer of beadless pearls is thicker than that of beaded ones, and the structure and characteristics of nacre are the decisive factors for pearls grading. The constituent of nacre is CaCO_3_ of which polymorphs may be aragonite, vaterite and calcite^11^. Unlike the vaterite, which is an unstable phase of CaCO_3_ and is rarely detected in Chinese fleshwater peals, aragonite is the main phase of CaCO_3_ in these pearls. During the process of growth, the nacre layer of beadless pearls may generate the crack fulfilled with the calcite^12^. The calcite, mixing with other organic matters, appears yellow and lows the quality of pearls. Therefore, it is critical to establish a non-destructive and efficient method for internal quality assessment of beadless freshwater pearls.

The subject of this study is to investigate whether the OCT technique is appropriate for internal classification. With the surface method like imaging, the internal classification technique lays on the foundation of the online classification. When OCT applied to freshwater pearls, classification speed becomes particularly important. Each mother oyster can cultivate dozens of pearls, and manual checking batches of pearls from their OCT image is a time consuming work. To speed up the classification process and develop online device, proposing automated grading method based on OCT images become more emergent.

Over recent decades, the utilizations of computer vision technique on OCT images concentrate on medical filed^13^,^14^. Most applications focus on monitoring the health status of vivo tissue, such as eye, arteria coronaria, esophagus, gastrointestinal tract. A small number of methods have been proposed to detect the ocular diseases^15^,^16^,^17^,^18^. But the automated classification or diagnosis and feature extraction still stays in primary research directions, and they were also the key problems need to be solved in pearls classification. In terms of automated measurement, Ming and Sun present automated thickness measurement methods of nacre for seawater pearls^19^. Most of seawater pearls are beaded, and the nacre thickness is the primary pearls evaluating indicator. Whereas, most of fleshwater pearls are beadless, the structure of nacre layer is the key factor to internal quality. Based on related reports, we attempt to classify the ZhuJi freshwater pearls by OCT internal inspection, meanwhile, to propose an automated algorithm to identify the crevice, cracks, or blemishes filled with calcite in nacre layer. By employing an integrated scanning galvanometer for scanning the whole round structure, the pearls with or without structural defects in nacre layer could be classified.

## Results

### Inspection of the internal defect of pearl

**Figure 1:**
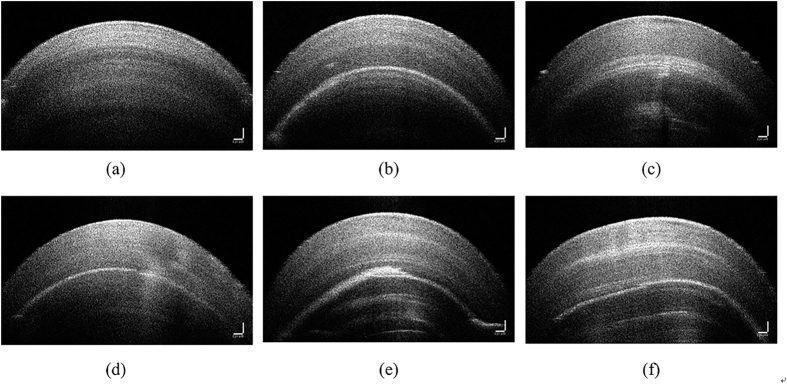
OCT images of beadless Chinese ZhuJi fleshwater pearls that retained no defects and cracks (**a**), and the rest (**b–f**) contained internal defective sub-layer with various shape, angel and depth.

The beadless freshwater peal was wrap up by nacre that built up in layers. Figure 1 shows the OCT image of pearl samples with normal nacre layer (a) and with defective sub-layers (b)–(f). Chinese ZhuJi fleshwater pearls were prone to generate silts or plaques fulfilled with calcite or other organic matters. Lustre and transparency were key parameters of pearl quality, and they were decided by the reflection, interfere and refraction when light penetrated the nacre sub layers. Crystalline and alignment of aragonite crystal were related to the lustre and transparency, in principle, high crystallinity and ordered alignment meant higher values of pearls. But the calcite layer always mixed with organic composition that lowed the reflection of light. Excessive organic composition was the cause of irregular structure and caused the scatter of light. The OCT image was based on light interfere and revealed micromorphology and microstructure of pearls. Besides the inspection of internal structure of nacre, the OCT images could be used for detecting the defect layers. As shown in Fig. 1, the normal sample consisted of sub layers as the annual rings. The samples from Fig. 1(b–f) had amorphous crevices and blemished in nacre layer with larger pixel grayscale. From visual presentation of those samples in OCT image, we drew a conclusion that the OCT technique could identify the internal defect of beadless pearls.

### Automatic detection of defect layer

**Figure 2:**
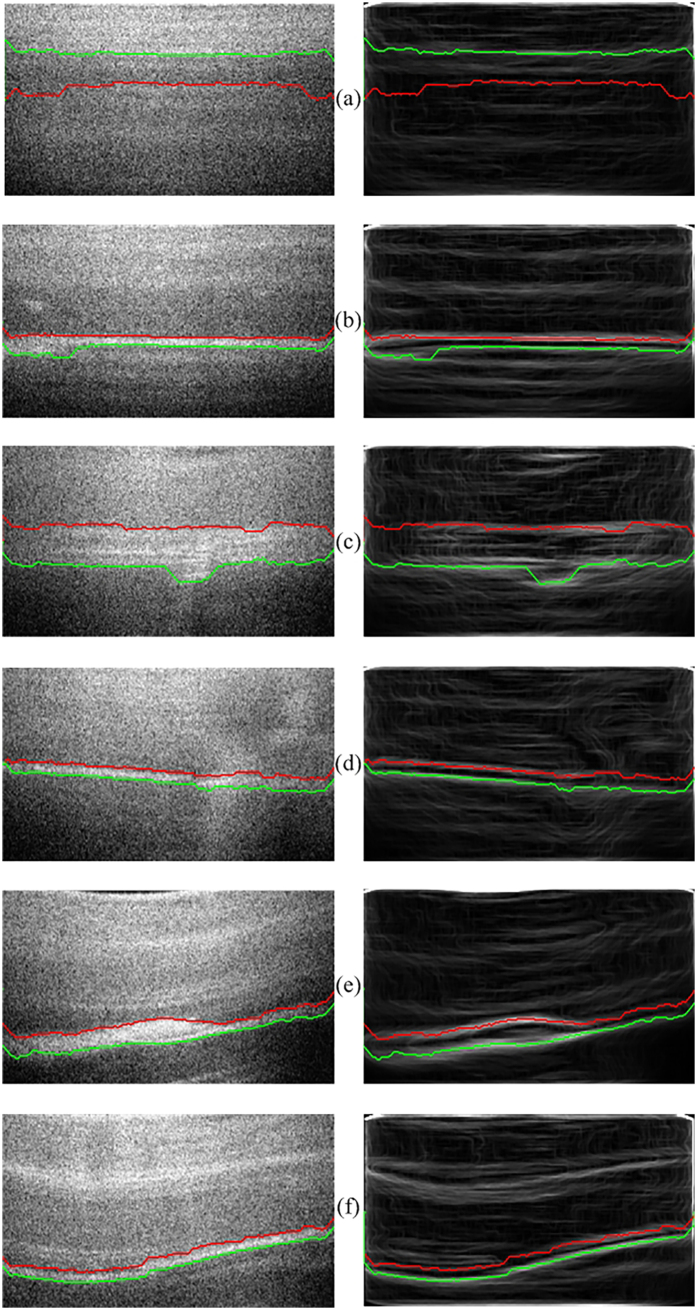
Result of defective sub-layer segmentation of six typical samples: (**a**) no defects and cracks (**a**), and (**b–f**) contained internal defective sub-layer. Red line: up boundary; Green line: down boundary. Images in left column: flattened images. Images in right column: vertical gradient of flattened images.

To assess the performance of our graph segmentation algorithm and its ability to detect the up and down boundaries of defective layer, we inspected the OCT images of 5 typical samples that were same as samples described in Fig. 1(b–f). Figure 2(b–f) shows the estimated boundaries of these samples. The defect sublayer, in general, was a stripe area, but its shape was various. The advantage of graph method was that it overcomes the influence of shape, bending and jump of the sub-layer which located at boundaries. From both original images of samples with defective sub-layers and their vertical gradient image, it was proved that the graph method could extract the defect area in nacre layer of beadless pearls with different morphological characteristics. The sample showed in Fig. 2(a) was normal pearl without any defects and cracks, but the graph method still estimated the boundaries. Even Fig. 2(a) shows the down boundary is above the up boundary. Generally, the pixel intensity in pearl OCT image decreased from top to bottom. The down boundary (green line in Fig. 2), being defined by light-to-dark change, emerged in the junction between first and second ring round at upper part of image. On the contrary, the up boundary, representing the dark-to-light, appeared at the junction between second and third ring round.

### Fully automated Pearls internal defect classification

Total 30 pearls samples were provided by Ruanshi company, one of the biggest fleshwater pearls manufacturer in Zhuji City, China. All the samples were beadless pearls harvested at different season in year 2015. Pearls were divided into two grades, with or without internal defective sub-layer. In defect set of samples, the samples contained different forms of cracks filled with calcite. To propose a fully automated algorithm for pearl classification, we needed to use the segmentation method on OCT images with or without defective sub-layers. The pixels/nodes around the boundary were significantly distinct between normal and defective sub-layers. From the flattened images (left column in Fig. 2), the pixels intensity change on both sides of boundary in defective sub-layer was much stronger than that in normal ring-shaped sub-layer, which caused differential vertical gradient of pixels on the boundaries. The vertical gradient of boundary pixel of defective sub-layer was greater than that of normal layer, and it corresponded to the grayscale of vertical gradient images where the higher brightness on the boundary of defective sub-layer was observed on the right column in Fig. 2. So the average vertical gradients of nodes/pixels in boundaries were figured out and listed in Table 1. From the Table 1, we reached the same conclusion that the absolute value of vertical gradient of defective sub-layer was larger than that of normal layer, and proper gradient threshold could be applied to classify two kinds of layer, even could classify pearls structure situations. We set the threshold of up boundary and down boundary at 180 and −240 respectively for classification task, and reached 100% classification accuracy for all 30 pearl samples.

The automated classification algorithm was programed in MATLAB (The MathWorks, USA) installed on a personal computer with I7-4710 CPU, 12GB RAM and Windows-7 operation system. The average classification time for one OCT image was about 4 seconds. The step of segmentation costed more than 95% of operational time, and running time was dramatically cut when using smaller fields-of-view. The horizontal and vertical space resolution of current setting of OCT system were 6.73 and 3.48 μm per pixel respectivly, and they were quite beyond the requirements of inspection of defective sub-layer. In the resize step, we tried to reduce the cropped image to smaller size with the help of interpolation. When quarter section of flattened image was regarded as the input of segmentation, the classification time was reduced to less than 1 second. That would be benefit for fast or on-line classification of internal quality of pearls.

We continued to collect extra 30 samples to verify the stability of automated algorithm, and the classification correctness of validation set was 100%, which proved the effectiveness of proposed algorithm. From validation samples and their OCT images, the vertical gradient ranges of normal and defect pearls were noticeable different. Therefore, the threshold decision ensured minimal condition impact.

## Discussion

In pearl industry, there had three kinds of non-destructive method to detect calcite-sublayers: (1) artificial visual detection by expert experience, (2) micro computed tomography (CT), (3) OCT method. The artificial visual detection was not a reliable method, as it highly depended on human judgment. Due to development of photo-electricity test technology, several other techniques had introduced to determine the pearl’s internal quality, such as spectroscopy and microscope^20^,^21^. X-ray computed micro tomography was regarded as a useful tool for identifying nature or cultured pearls, and had been applied to gain insight into pearl internal structure^22^. Many research groups distinguished natural pearls from beaded and non-beaded cultured pearls based on computed x-ray micro tomography which revealed the internal structure at micrometer-scale resolution. However, the x-ray technique had notable drawback which caused the pearls tarnished after long time inspection, and the direct consequent was value losing. The microscope only got the cut off images and its depth resolution can’t meet the demand of internal inspection. OCT technique was regarded as a new approach in pearl industry. After acquiring the OCT image, most of reported application relied on the visual check or morphological analysis, which are time-consuming and inefficient work. We presented a novel method for internal classification of beadless Chinese Zhuji fleshwater pearls by using OCT images. This method fully automated identified the defect structure/sub-layers. The proposed method relied on the vertical gradient changes of the flattened image, and was adapted to defected layers with different shape, curvature and thickness. The grading utilized the average gradient difference between normal and defective layers. The proposed method, as a supplementary for OCT application in pearl analysis, effectively distinguished the structural imperfection in nacre layer of beadless pearls, and complicated the classification task. Moreover, our method achieved perfect sensitivity and high specificity. Combined with surface evaluation like image, it laid the foundation of on-line automated classification beltline, which can be promoted to other product classification.

The graph segmentation method also gave the reference to automated thickness measurement of nacre of beaded pearls^9^,^19^. Nacre thickness was one of the key quality parameter of beaded pearls, and several automated measurement methods were reported. In these methods, edge operator or pixel classification was carried to extract the lower boundary. We believed that graph approach had better performance than other existing approaches. However, the study of such case was out of the scope of paper.

## Methods

### OCT system description

Spectral domain OCT system(TELSTO 1300V2, thorlabs, USA) was used in our experiment. The OCT system consisted of light source, isolator, coupler, spectrometer, computer, interferometer, scanning mirror and lens, where the interferometer, galvo-scanning mirror and lens were integrated in rigid scanner (OCTG, thorlabs, USA), and the rest parts were assembled in a base unite. The scanner was mounted in a custom-built stand with motorized rotation stage. The OCT images were acquired and managed by ThorImage Software installed in computer (V4.2, thorlabs, USA). Before the acquisition, background was calibrated and reference intensity was manually adjusted at optimal settings. In order to increase the sensitivity of the OCT image, the integration time of spectrometer maintained in minimal with consequent acquisition speed at 5.5 kHz. The sizes of A-scan and B-scan were both 1024 pixels that generated the 1024*1024 size OCT image. The filed-of-view was 7*3.57 mm, and each pixels in A-scan and B-scan occupied 3.48 and 6.73 μm. The high resolution of OCT image had benefit for inspecting the internal structure of pearls.

### A generalized pearls grading algorithm

**Figure 3:**
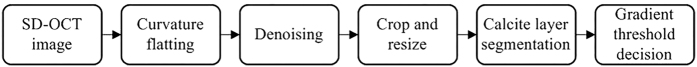
The generalized algorithm schematic.

After acquiring the OCT images of pearls, the task of classification algorithm was to detect the calcite layer and make the classification decision. The feature of the calcite sub-layer was interference intensity increasing, which was larger than that of normal nacre sub-layer and presented lighter area in image. Hence, the main step of algorithm was shifted to identify the calcite layer. The core steps of proposed algorithm are described in Fig. 3.

### Back ground segmentation, flattening and denoising

One OCT image consisted of upper and downer parts, the background and pearl target. The following steps were focused on the target part, and the first step became segmentation of this part. From prior knowledge base, the background was contaminated by speckle noise, so the denoising helped the segmentation procedure^23^. The mean (*μ*) and variance (*σ*) of grayscales of top 10 rows in OCT images (we had confirmed that 10 rows of all the images were background part) were calculated for threshold denoising with empirical threshold as *μ* + 7*σ.

Then edge detection based on Canny operator was carried out to find the edge between target and background. Canny edge detection was a widely used technique to extract the edge between pearl target and background in OCT image. The Canny edge detection algorithm consisted of five main steps, including smoothing and removing noise by Gaussian filter, intensity gradients searching, getting rid of spurious response by non-maximum suppression, determining potential edges by double threshold, detection of edges by suppressing. Among the edge detection methods, canny edge detection algorithm was one of the most strictly defined methods that provided reliable edge in OCT image.

**Figure 4:**
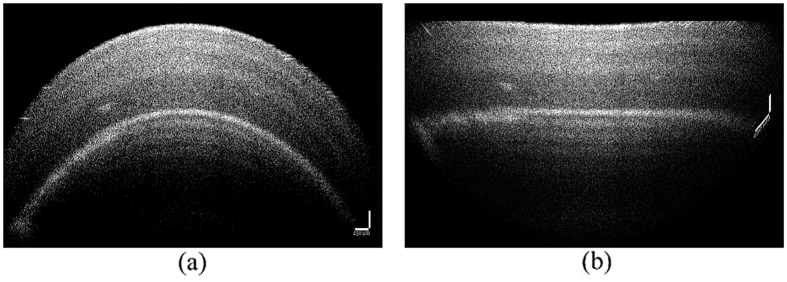
OCT image flattening (**a**) original image (**b**) flattened image.

Following, due to unsmooth of detected edge, and to reduce the perceived pearl curvature, the pearl curvature was required to be flattened. The polynomial fitting was used to fit the unsmooth edge, and shifted the point in target part up and down, and made all the points of edge laid on a horizontal line. Figure 4 demonstrated flattening process, where Fig. 4(b) was the version of Fig. 4(b) with the target curvature flattened.

There had bright and dark spots in OCT images caused by the speckle and noise in data acquisition, and they were removed by speckle averaging with reference to the surrounding pixels. Even applied the method provided by the ThorImage software, the level of speckle noise still affected the classification process. A [10*40] median filter smoothed the image with small impact on ring-shaped boundary^24^.

### Image crop and resize

After flattening, the marginal of whole image became blurring, and it was cut before layer segmentation. We cropped 600*300 pixels from flattened image. In horizontal direction, 600 rows were from fitted edge to bottom of image. In vertical direction, 300 columns were around the center of image. To accelerate the image process speed for online application, we also reduced the cropped image to the half with nearest-neighbor interpolation, and avoided detail loss of nacre layer by interpolation process.

### Calcite layer segmentation

From the flattened image, the calcite defect took on the layered structure, so the grading task was converted to layer segmentation. The nacre layer of beadless pearls was formed like tree ring, such architecture feature was regarded as horizontal multilayers. However, the calcite layer was different from other aragonite crystal layer where the intensity of interference was significantly stronger than that in normal nacre. Meanwhile, the grayscales of corresponding layer in OCT image were lager than other layers. The aim of segmentation was to find up and down boundaries of ‘light’ sub-layer and classify between normal and defective ones.

Graph segmentation was one of the effective approaches for OCT images, and had been applied into ophthalmic applications^25^,^26^. Graph segmentation considered pixels as nodes with links connecting the nodes. These links were defined as edges (this concept was only for graph segmentation and different from edge detection). The method was to find a pathway consisted with edges across the graph. After proposed the weights assignment and start/end note selection, the optimal path that travelled across the graph from a start node to an end node was the route in which the sum of weight was at a minimum level. There were different routes connecting two points where each route (a set of edges) had individual total sum of weights^27^, and the crucial issues of optimal route searching was the edge weights assignment.

Because of the uncertainty of geometry, only grayscale intensity difference represented the layered feature. After median filtered, the image became smooth with neighboring pixels. Therefore, each node had eight edges connecting with neighboring nodes, and nodes outside neighboring field were supposed to be disconnected. To find the up and down boundaries of calcite layer, the gray discontinuous area should be assigned lower weights. In flattened pearl OCT images, the defective sub-layer showed the approximate horizontal structures told apart by grayscale intensity changes in the vertical direction. The vertical gradient reflected these changes and the weights between nodes defined as follows:
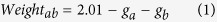
where *Weight*_*ab*_ was the weight corresponding to edge between nodes *a* and *b*, *g*_*a*_ and *g*_*b*_ were the normalized vertical gradient of nodes *a* and *b*. In boundary area, the grayscale changed dramatically that indicated the higher gradient close to 1. On the contrary, the weights decreased towards 0.01. The edges with lower weighs were searched by graph segmentation methods. Nonetheless, the polarity of gradients on up layer and down boundaries were different. The up boundary had dark-to-light changes, the gradients were positive values. Conversely, the down boundary had light-to-dark changes, the gradients were negative values. For the dark-to-light and light-to-dark transition, the positive and absolute values of negative gradients were used for up and down boundaries, respectively. To enlarge vertical gradient on boundaries, we employed power-law transformation defined as S = C*γ^r to increase the dynamic range of grayscale of images, where r and s were input and output gray levels. For pearl application, C = 1.2 and γ = 1.5 were selected.

Before the starting of segmentation, the start and end notes were required by manual selection. For automatic grading, we utilized a strategy proposed by Stephanie that omitted the start point assignment^28^. As the graph segmentation selected the path with minimum weights, we added two extra columns of nodes to left and right sides of OCT image, and also assigned 10^−5^ as the weights of edges connecting with added nodes. The weights of added notes were significantly smaller than that in original image which were at least greater than 0.01 from equation 1. Hence the added nodes were considered as almost connected, and the estimated boundary passed through these nodes with extremely low resistance. The added nodes and weights made it possible to assign any nodes in left added row as start point and those in right added row as end point, and the boundary lateral passed the whole image through edges with minimum sum of weights. We assigned the middle vertical points in two added rows as start and end point, and used the Dijkstra’s algorithm (one of the classic graph approach) to find the boundaries^29^.

### Gradient threshold decision

As mentioned above, the nacre of beadless freshwater pearl formulated with annual ring structure. Even the pearl without any defect, caused by ring effect, the graph segmentation method still could find the boundaries in OCT image. After analysis of all nodes/pixels around up and down boundaries, we found that absolute vertical gradients of boundary nodes of defective sub-layers were obviously larger than those of normal samples. The ring boundary presented the slim and dim lines was totally different from defective sub-layers that presented irregular light strips in OCT images. The threshold of gradient was set up for decision between normal and defective sub-layer. In order to avoid disturbance of random noise, the average gradient of boundary pixels was compared with thresholds.

## Additional Information

**How to cite this article**: Zhou, Y. *et al*. Automated Internal Classification of Beadless Chinese ZhuJi Fleshwater Pearls based on Optical Coherence Tomography Images. *Sci. Rep.* **6**, 33819; doi: 10.1038/srep33819 (2016).

## Figures and Tables

**Table 1:** Average vertical gradient of boundary pixels.

Up boundary		Down boundary	
Normal	Defect	Normal	Defect
104.8	292.3	−169.0	−343.6
