# Corrigendum: Automated Internal Classification of Beadless Chinese ZhuJi Freshwater Pearls based on Optical Coherence Tomography Images

**DOI:** 10.1038/srep40270

**Published:** 2017-02-23

**Authors:** Yang Zhou, Tiebing Liu, Yang Shi, Zhengwei Chen, Jianwei Mao, Wujie Zhou

Scientific Reports 6: Article number: 3381910.1038/srep33819; published online: 09 26 2016; updated: 02 23 2017

In the original version of this Article, all instances of “freshwater” were incorrectly given as “fleshwater”. This error has now been corrected in the PDF and HTML versions of the Article.

